# COVID-19 Vaccination and Cancer: Correspondence

**DOI:** 10.31557/APJCP.2021.22.11.3417

**Published:** 2021-11

**Authors:** Rujittika Mungmunpuntipantip, Viroj Wiwanitkit

**Affiliations:** 1 Private Academic Consultant, Bangkok Thailand.; 2 Honorary Professor, Dr DY Patil University, Pune, India.

**Keywords:** COVID-19, vaccination, cancer

**Dear Editor**

Dear Editor, we would like to correspond on “COVID-19 Vaccination and Cancer, the Need for more Data (Javadinia et al., 2021).” Javadinia et al., (2021) mentioned for four important points for further researches regarding COVID-19 vaccination and cancerous cases. Four points are efficacy, protectivity, immunogenicigity and interference by cancer therapy (Javadinia et al., 2021). We agree that vaccine is required for patient with underlying cancer and new data usually confirm for advantage of vaccination. Nevertheless, we would like to add more item to be focused. The important item is on adverse effect of vaccination. In general, vaccine is considered safe but there should be specific precaution in some specific cases. For example, some cancers might have underlying high blood viscosity. Inducing of increased blood viscosity after vaccination is possible (Joob and Wiwanitkit, 2021). If vaccine is applied, there might be an excessive high blood viscosity and causes problem (Mungmungpuntipantip and Wiwanitkit, 2021). 

Specific protocol for monitoring of adverse effect is required for management of cancerous patient and it will be useful preventive measure against unwanted adverse effect of vaccination.
